# Ultra-high-field arterial spin labelling MRI for non-contrast assessment of cortical lesion perfusion in multiple sclerosis

**DOI:** 10.1007/s00330-018-5707-5

**Published:** 2018-10-02

**Authors:** Richard J. Dury, Yasser Falah, Penny A. Gowland, Nikos Evangelou, Molly G. Bright, Susan T. Francis

**Affiliations:** 1 Sir Peter Mansfield Imaging Centre, School of Physics and Astronomy, University of Nottingham, University Park, Nottingham, NG7 2RD UK; 2 Clinical Neurology, Division of Clinical Neuroscience, School of Medicine, University of Nottingham, Queen’s Medical Centre, Nottingham, NG7 2UH UK; 3 Physical Therapy and Human Movement Sciences, Feinberg School of Medicine, Northwestern University, 645 N. Michigan Avenue, Suite 1100, Chicago, IL 60611 USA; 4 Biomedical Engineering, McCormick School of Engineering, Northwestern University, Evanston, IL 60208 USA

**Keywords:** Magnetic resonance imaging, Perfusion, Multiple sclerosis, Grey matter

## Abstract

**Objectives:**

To assess the feasibility of using an optimised ultra-high-field high-spatial-resolution low-distortion arterial spin labelling (ASL) MRI acquisition to measure focal haemodynamic pathology in cortical lesions (CLs) in multiple sclerosis (MS).

**Methods:**

Twelve MS patients (eight female, mean age 50 years; range 35–64 years) gave informed consent and were scanned on a 7 Tesla Philips Achieva scanner. Perfusion data were collected at multiple post-labelling delay times using a single-slice flow-sensitive alternating inversion recovery ASL protocol with a balanced steady-state free precession readout scheme. CLs were identified using a high-resolution Phase-Sensitive Inversion Recovery (PSIR) scan. Significant differences in perfusion within CLs compared to immediately surrounding normal appearing grey matter (NAGM_local_) and total cortical normal appearing grey matter (NAGM_cortical_) were assessed using paired t-tests.

**Results:**

Forty CLs were identified in PSIR scans that overlapped with the ASL acquisition coverage. After excluding lesions due to small size or intravascular contamination, 27 lesions were eligible for analysis. Mean perfusion was 40 ± 25 ml/100 g/min in CLs, 53 ± 12 ml/100 g/min in NAGM_local_, and 53 ± 8 ml/100 g/min in NAGM_cortical_. CL perfusion was significantly reduced by 23 ± 9% (mean ± SE, *p* = 0.013) and 26 ± 9% (*p* = 0.006) relative to NAGM_local_ and NAGM_cortical_ perfusion, respectively.

**Conclusion:**

This is the first ASL MRI study quantifying CL perfusion in MS at 7 Tesla, demonstrating that an optimised ASL acquisition is sensitive to focal haemodynamic pathology previously observed using dynamic susceptibility contrast MRI. ASL requires no exogenous contrast agent, making it a more appropriate tool to monitor longitudinal perfusion changes in MS, providing a new window to study lesion development.

**Key Points:**

• *Perfusion can be quantified within cortical lesions in multiple sclerosis using an optimised high spatial resolution arterial spin Labelling MRI acquisition at ultra-high-field.*

• *The majority of cortical lesions assessed using arterial spin labelling are hypo-perfused compared to normal appearing grey matter, in agreement with dynamic susceptibility contrast MRI literature.*

• *Arterial spin labelling MRI, which does not involve the injection of a contrast agent, is a safe and appropriate technique for repeat scanning of an individual patient.*

## Introduction

Cortical lesions (CLs) in patients with multiple sclerosis (MS) are associated with physical disability [1] and cognitive impairment [2]; however, little is known about the formation and development of CLs due to their typically small size [3] and the insufficient sensitivity and spatial resolution offered by conventional imaging modalities. Emerging techniques using ultra-high-field magnetic resonance imaging (MRI) can significantly improve the detection of CLs compared to 3 Tesla (T) scanners [4], and can advance our understanding of how these lesions develop or resolve over time [5].

Techniques including phase-sensitive inversion recovery (PSIR) [6] and double inversion recovery (DIR) [7] can identify CLs and track their structural development. However, it is also desirable to understand changes in physiology that may precede or dictate these overt structural changes. To date, the haemodynamic changes within CLs have been characterised using dynamic susceptibility contrast (DSC) MRI, revealing that local haemodynamics change with the status of the lesion: chronic CLs in grey matter exhibit reduced perfusion and cerebral blood volume [8–10], whereas in acute lesions elevated cerebral blood volume has been reported [8]. Reports of changes in perfusion prior to lesion formation and contrast enhancement [11] suggest that studies of local haemodynamics may predict such tissue damage, identifying a critical window for intervention.

To characterise rapid changes during periods of disease activity and CL development, patients must be scanned repeatedly and frequently. DSC MRI, although the clinical gold-standard for assessing haemodynamic changes, is not well suited to such longitudinal studies. Concerns over dose-dependent deposition of gadolinium-based contrast agents in the brain caution against frequent repeated exposure [12–15].

Arterial spin labelling (ASL) MRI provides an alternative method for quantifying local tissue perfusion that requires no injection of exogenous contrast. ASL has been successfully used to measure perfusion deficits in cortical grey matter in early stages of MS compared to healthy controls, demonstrating its clinical sensitivity [16]. CL perfusion has been examined using Pseudo-Continuous ASL at 3T and DSC data collected to validate the results: there was poor agreement in perfusion quantification between the methods, highlighting the challenges in making robust ASL perfusion measurements in small CLs compared to larger regions, especially at coarser spatial resolution. The inherently low contrast-to-noise ratio of ASL techniques is substantially improved when scanning at ultra-high-field (here defined to be 7T) [17], allowing data to be collected at higher spatial resolution. However, there are numerous challenges when performing ASL at 7T that require optimisation of the imaging protocol [18]. In this study, we assess the feasibility of using an optimised, ultra-high-field, high-spatial resolution, low-distortion ASL acquisition [19] to quantify perfusion in chronic CLs, demonstrating proof of concept that this technique is suitable for characterising local haemodynamic pathology in MS.

## Materials and methods

This cross-sectional prospective study was approved by the local research ethics authority. Twelve patients with MS were recruited after giving informed consent. All patients were selected based on having known pre-existing chronic CLs from scans of two prior studies [6, 20]. One of these studies developed the PSIR technique for improved CL detection [6] and the second examined the ‘central vein sign’ in white matter lesions [20]; neither of these earlier studies acquired ASL data or quantified perfusion. Both studies completed prior to the end of December 2014. Access to these earlier scans followed ethical guidelines to which consent was given. All scanning for this study took place between September and December 2015, at least 8 months after identification of CLs. As such, all CLs in this study are considered chronic.

The 12 recruited patients included seven relapsing-remitting and five progressive (four primary progressive and one secondary progressive) MS patients (eight male, four female; mean age 50 years (range 35–64); median Expanded Disability Status Scale 4.5 (range 2.0–6.0)).

### Magnetic resonance acquisition

MRI was performed on a 7T Philips Achieva scanner (Philips Medical Systems, Best, The Netherlands) using a 32-channel receive coil. Details of acquisition parameters are provided in Table 1. To identify CLs, a whole-head PSIR scan was performed using a tailored adiabatic inversion pulse to ensure efficient inversion in areas of radio frequency (RF) inhomogeneities found at ultra-high field [21]. The timings of the turbo field echo (TFE) readouts were optimised to suppress the signal from voxels containing equal amounts of grey and white matter, thus producing a clear observable boundary between them [6]. The PSIR images were acquired at high spatial resolution (0.6 mm isotropic) to minimise partial volume effects in small CLs, allowing accurate boundary detection.

**Table 1:** Scan acquisition parameters. Acquisition parameters for the Phase Sensitive Inversion Recovery (PSIR) 3D turbo field echo (TFE) scan, and the single and multi-phase arterial spin labelling (ASL) scans with a balanced steady-state free precession (bSSFP) read-out

Parameter	PSIR	Single-phase ASL	Multi-phase ASL
Readout	3D-TFE	bSSFP	bSSFP
Field of view (AP, RL, FH)	200 × 181 × 120 mm^3^	192 × 192 × 3 mm^3^	192 × 192 × 3 mm^3^
Voxel size (AP, RL, FH)	0.6 × 0.6 × 0.6 mm^3^	1.2 × 1.2 × 3.0 mm^3^	1.2 × 1.2 × 3.0 mm^3^
Echo time (TE)	6 ms	1.9 ms	1.9 ms
Repetition time (TR)	13 ms	3.8 ms	3.8 ms
Flip angle	8°	50°	35°
SENSE (AP, RL, FH)	2, 1, 2	1, 2.5, 1	1, 2.5, 1
Post-labelling delay (PLD)	-	1,400 and 1,800 ms	200, 550, 900, 1,250, 1,600, 1,960, 2,300, 2,650 ms
Averages	1	50	40
Scan duration (mm:ss)	12:55	5:06 per PLD	6:40 all PLDs

ASL data were acquired using a flow-sensitive alternating inversion recovery (FAIR) ASL scheme with in-plane pre-saturation using a WET (Water suppression Enhanced through T1 effects) scheme and a sinc post-saturation pulse. A balanced steady-state free precession (bSSFP) readout was used to achieve high spatial resolution (1.2 × 1.2 × 3.0 mm^3^) with minimal distortions, essential for accurate co-registration of small CLs. A single axial imaging slice was positioned to transect one or more CLs identified from the PSIR acquisition. Single-phase ASL data were collected at post-labelling delay (PLD) of 1,400 and 1,800 ms to determine tissue perfusion (50 label-control pairs were acquired at each PLD, bSSFP readout collected using α/2 pulse at a time TR/2 before a train of RF pulses with α of 50^o^ to reach a steady state). In addition, multi-phase ASL data were acquired using a Look-Locker bSSFP readout (with flip angle α of 35°) comprising eight PLDs of 200, 550, 900, 1,250, 1,600, 1,960, 2,300 and 2,650 ms (40 label-control pairs, to estimate arterial transit time and locate intravascular signal contributions). An M_0_ image was acquired for both single- and multi-phase bSSFP acquisitions to allow absolute perfusion quantification.

### Cortical lesion identification

CLs were identified from the PSIR modulus image during the scanning session by a trained rater (YF) in order to position the ASL slice appropriately. CLs were selected as any hypointense demarcated lesions within the cortical ribbon, as described by Mougin et al [6]. Masks were drawn around the CLs using MIPAV (Medical Imaging Processing Analysis and Visualisation, CIT, NIH, Bethesda, MD, USA).

### Image co-registration

The ASL and PSIR sequences were acquired back-to-back, to minimise misalignments between these data acquisitions. However, in three patients there was significant movement between the ASL and PSIR scans. In order to accurately locate the CLs in ASL data-space, the PSIR data were co-registered to the ASL data. Due to the limited coverage of the ASL scan, automated co-registration techniques were not appropriate. Instead, the PSIR slices corresponding to the ASL slice were identified and corrective rotations were applied manually as needed using FSL FLIRT [22]. The CL masks were then co-registered to the ASL data by applying these transformations.

### ASL pre-processing

The ASL data were brain extracted and motion corrected using 2D in-plane co-registration using FSL MCFLIRT [23], and any label-control pairs that contained > 1.2 mm translational movement (the in-plane resolution of the ASL voxels) were discarded. The label images were subtracted from the corresponding control images, and these difference images were averaged for each PLD using a Huber M-estimator to remove outlier signals and generate robust perfusion-weighted images [24].

### Perfusion quantification

The base M_0_ images were co-registered to the average ASL label image using FSL FLIRT. A T_1_ map was obtained by fitting the label images from the multi-phase ASL data to a Look-Locker saturation recovery curve:

$$ S={M}_0\left(1-\alpha \bullet \exp \left(-\frac{t}{T_1^{\ast }}\right)\right) $$

where M_0_ is the equilibrium magnetisation, *α* is the saturation efficiency, *t* is the time following saturation and T_1_* is the apparent longitudinal relaxation. The true T_1_ can then be calculated from the T_1_* using:

$$ \frac{1}{T_1}=\frac{1}{T_1^{\ast }}+\frac{\ln \left(\mathrm{cos}\upalpha \right)}{\tau } $$

where α is the flip angle and *τ* is the spacing of the Look-Locker readouts (350 ms). The T_1_ of the arterial blood was estimated by fitting an inversion recovery to the signal in the sagittal sinus and correcting for oxygenation [25].

The multi-phase ASL data were fit to the model described by Francis et al [25] to produce a transit time map. This method also fits for perfusion; however, the SNR of the multi-phase ASL data is considerably lower than that of the single-phase ASL data [26]. Thus, this transit time map, the T_1_ map, and the fitted value for the T_1_ of arterial blood were used with the averaged single-phase data in a model fit, as described by Gardener et al [17], to quantify perfusion in ml/100 g tissue/min. To optimise this fit, the starting parameters were chosen by comparing the voxel-by-voxel ASL signal to a lookup table of modelled ASL signals. Parameters producing the best estimate (assessed using the sum of square differences) were used to initialise a Nelder-Mead simplex direct search implemented in MATLAB (The MathWorks, Inc., Natick, MA, USA).

CL perfusion was calculated by averaging the voxel-wise perfusion values inside the CL mask. A mask of local normal appearing grey matter (NAGM_local_) was created by dilating the CL mask to a radius of 12 mm, then restricting this to a grey matter mask obtained from segmenting the PSIR image using FSL FAST. The mean perfusion value inside this region, with the CL removed, was defined as NAGM_local_ perfusion. Finally, the average perfusion in total cortical normal appearing grey matter (NAGM_cortical_) was also computed.

### Lesion eligibility

Any CL with a volume of less than three ASL voxels (12.96 mm^3^) was discarded. As vascular crushing could not be applied in this bSSFP ASL acquisition, fitted perfusion values were influenced by both perfusion and intravascular blood flow contamination in the voxel. A histogram of NAGM_cortical_ perfusion values was created and fitted to a mixture model of Gaussians (allowing for multiple undetermined perfusion and inflow signal sources); any voxel containing a ‘perfusion’ peak value of five times greater than the NAGM_cortical_ perfusion peak was assumed to be dominated by vascular inflow, and this was used to create a vascular inflow mask. CLs overlapping with this mask were discarded.

### Statistical comparison of CL and NAGM perfusion

Paired two-sample t-tests were used to determine differences in perfusion (significance threshold *p* < 0.05). Statistical analyses were performed using Minitab 17 Statistical Software (2010) (State College, PA, USA: Minitab, Inc. (www.minitab.com)).

## Results

Data from two participants were discarded due to head movement that moved CLs out of the ASL imaging volume. All remaining acquisitions were of sufficient quality to extract perfusion maps.

### Cortical lesion identification

**Fig. 1:**
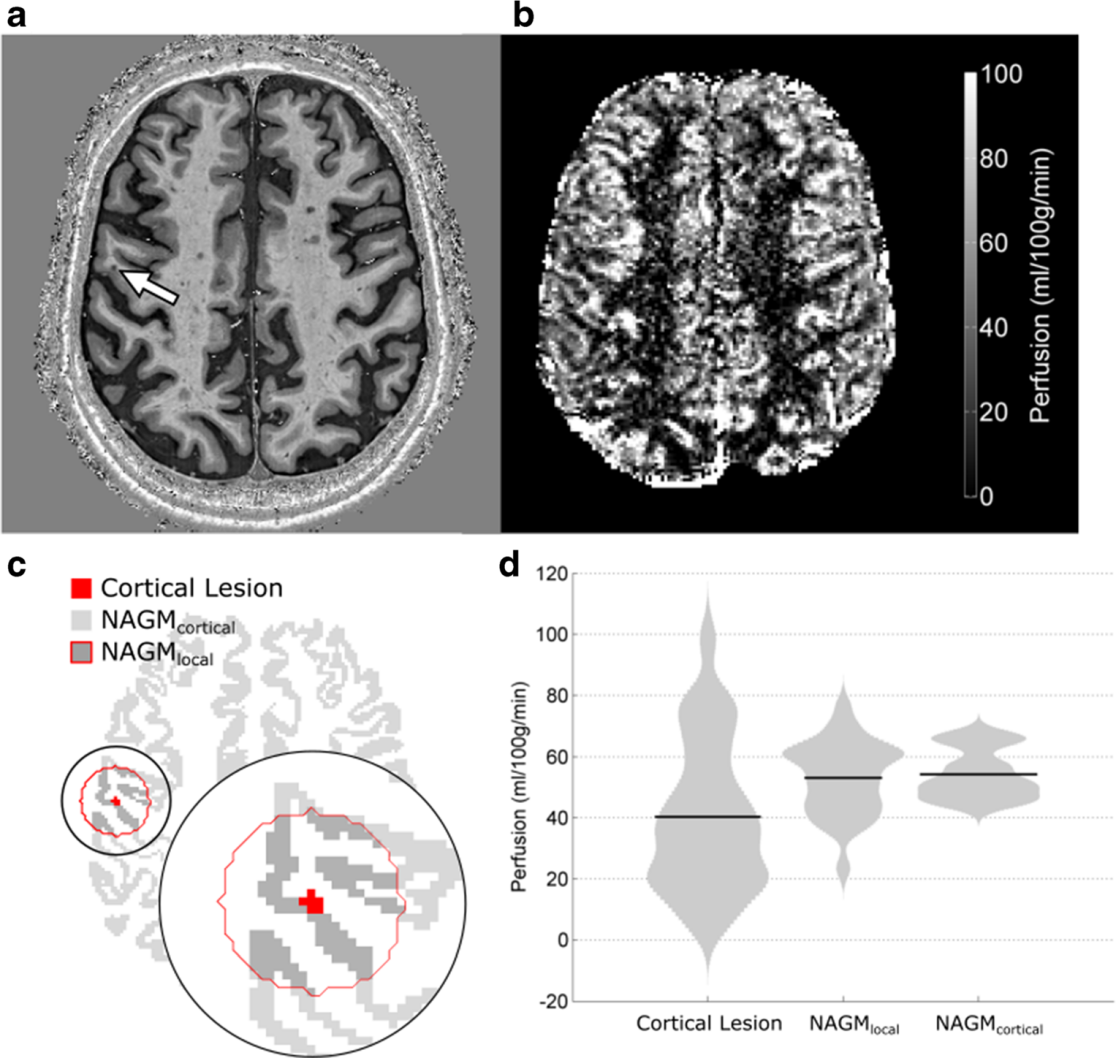
**a** Axial phase sensitive inversion recovery (PSIR) slice containing a cortical lesion (CL) indicated by the arrow. **b** Arterial spin labelling (ASL) perfusion map (ml/100 g/min) of slice shown in **a**. **c** Binary mask of the cortical lesion, local and cortical normal-appearing grey matter (NAGM_local_ and NAGM_cortical_). **d** Violin plot of perfusion within CLs, NAGM_local_ and NAGM_cortical_ with the mean indicated (black line)

In total, 40 CLs were identified in the PSIR scans that overlapped with the ASL slice acquisition. After applying the eligibility criteria, 27 of the 40 lesions remained for analysis (67.5%); four contained vascular contamination (10%), eight were below the volume threshold (20%) and one failed both tests (2.5%) (Table 2). Examples of a PSIR image, perfusion-weighted ASL image, and CL, NAGM_local_ and NAGM_cortical_ masks are presented in Fig. 1a-c.

**Table 2:** Overview of identified cortical lesions (CLs)

Patient	CLs	Volume (mm^3^)
1	4	27.86†, 21.60†, 8.42*, 16.63
2	5	13.18†, 15.12, 47.09, 14.04
3	1	17.50
4	3	44.50, 17.50, 19.44
5	3	17.71, 30.02, 26.35
6	6	29.38, 8.64*, 17.93, 15.55, 6.48*, 21.17
7	4	22.03, 14.90, 25.49, 16.42
8	5	15.98, 15.34, 7.13*, 19.44, 15.98†
9	7	12.96, 7.56*, 8.86*, 8.86*, 8.42*, 14.26, 25.49
10	2	12.53*, 18.36

CLs (*) with a cortical volume less than 12.96 mm^3^ were discounted from analysis, along with those CLs (†) that had large vessel contamination

### Perfusion in CLs

The mean perfusion was 40 ± 24 ml/100 g/min in CLs, 53 ± 12 ml/100 g/min in NAGM_local_, and 53 ± 8 ml/100 g/min in NAGM_cortical_ (Fig. 1d). When comparing perfusion inside a given lesion to the associated NAGM_local_, CL perfusion was significantly lower, by 23 ± 9% (mean ± standard error, *p* = 0.013), and significantly lower than NAGM_cortical_ perfusion, by 25 ± 9% (mean ± standard error, *p* = 0.006).

## Discussion

To our knowledge, this is the first study measuring CL perfusion in MS using ASL at 7T. The majority of CLs showed hypoperfusion compared to NAGM, in line with the previous study by Peruzzo et al using DSC MRI at 1.5T [9], demonstrating that this optimised ultra-high-field ASL-MRI technique is sensitive to the expected pathological hypoperfusion.

This was an exploratory study aimed at using ASL with a bSSFP acquisition scheme to collect high spatial resolution data and minimal distortions, following on from its development in healthy subjects [19]. However, this restricted the acquisition to single-slice coverage, limiting our ability to robustly compare widespread cortical perfusion differences in patients compared to controls, and the clinical applicability of our technique. We are currently examining the reproducibility of 7T FAIR ASL with increased brain coverage using an alternative 3D-EPI acquisition scheme, for the comparison of perfusion measures in MS patients with controls. Using this imaging protocol in a test-retest study, we have recently demonstrated that 7T FAIR ASL cortical perfusion values are similarly repeatable in both MS patients and matched control subjects [27]. Based on this empirical evidence, we do not expect FAIR ASL data quality to be systematically impaired in studies of MS cohorts. In order to assess the repeatability of highly focal perfusion values (e.g. the spatial scale of CLs) in different control and patient cohorts, further technical development is needed to simultaneously achieve high-resolution data with whole-brain coverage by combining the bSSFP acquisition with simultaneous multislice (SMS) methods.

The ASL PLDs used here were optimised for quantifying perfusion in cortical grey matter, and both PLDs and spatial resolution would need to be adjusted to achieve sufficient contrast-to-noise ratio for robust quantification of white matter perfusion, which is significantly lower than NAGM and has been shown to be of the order of 16 ml/100 g/min [28]. Further generalisability of our observations is limited by the small sample size and heterogeneous disease course within our cohort. We plan to apply this new imaging protocol to study CL perfusion across the distinct MS subtypes to better understand variations in lesion development in this diverse patient group. Finally, previous work has reported poor agreement between ASL and DSC perfusion estimates in CLs at 3T [29], and we are now seeking to validate our perfusion results derived from ASL in MS patients with results obtained using DSC-MRI, the clinical gold-standard technique [27].

Unlike DSC, ASL requires no exogenous contrast agent, making it an appropriate tool to study dynamic perfusion changes in MS. ASL-MRI may facilitate the study of CL formation and development, to test new therapeutic strategies and better understand the heterogeneous disease course in MS.

## Abbreviations

ASL: Arterial spin labelling
CL: Cortical lesion
DSC: Dynamic susceptibility contrast
FAIR: Flow-sensitive alternating inversion recovery
MRI: Magnetic resonance imaging
MS: Multiple sclerosis
NAGM_cortical_: Normal appearing grey matter (cortical)
NAGM_local_: Normal appearing grey matter (local)
PLD: Post-labelling delay
PSIR: Phase sensitive inversion recovery
RF: Radio frequency
SNR: Signal-to-noise ratio

## References

[1] Calabrese M, Poretto V, Favaretto A (2012). Cortical lesion load associates with progression of disability in multiple sclerosis. Brain.

[2] Calabrese M, Agosta F, Rinaldi F (2009). Cortical lesions and atrophy associated with cognitive impairment in relapsing-remitting multiple sclerosis. Arch Neurol.

[3] Pitt D, Boster A, Pei W (2010). Imaging cortical lesions in multiple sclerosis with ultra-high-field magnetic resonance imaging. Arch Neurol.

[4] Nielsen AS, Kinkel RP, Tinelli E, Benner T, Cohen-Adad J, Mainero C (2012). Focal cortical lesion detection in multiple sclerosis: 3 Tesla DIR versus 7 Tesla FLASH-T2. J Magn Reson Imaging.

[5] Aphiwatthanasumet K, Mougin O, Geades N et al (2018) A longitudinal study of lesion evolution in multiple sclerosis using multi-contrast 7T MRI, Proceedings of the 26th Annual Meeting of the International Society for Magnetic Resonance in Medicine, Paris, France

[6] Mougin O, Abdel-Fahim R, Dineen R, Pitiot A, Evangelou N, Gowland P (2016). Imaging gray matter with concomitant null point imaging from the phase sensitive inversion recovery sequence. Magn Reson Med.

[7] Geurts JJ, Pouwels PJ, Uitdehaag BM, Polman CH, Barkhof F, Castelijns JA (2005). Intracortical lesions in multiple sclerosis: improved detection with 3D double inversion-recovery MR imaging. Radiology.

[8] Haselhorst R, Kappos L, Bilecen D (2000). Dynamic susceptibility contrast MR imaging of plaque development in multiple sclerosis: application of an extended blood-brain barrier leakage correction. J Magn Reson Imaging.

[9] Peruzzo D, Castellaro M, Calabrese M (2013). Heterogeneity of cortical lesions in multiple sclerosis: an MRI perfusion study. J Cereb Blood Flow Metab.

[10] Hojjat SP, Kincal M, Vitorino R (2016). Cortical Perfusion Alteration in Normal-Appearing Gray Matter Is Most Sensitive to Disease Progression in Relapsing-Remitting Multiple Sclerosis. AJNR Am J Neuroradiol.

[11] Wuerfel J, Bellmann-Strobl J, Brunecker P (2004). Changes in cerebral perfusion precede plaque formation in multiple sclerosis: a longitudinal perfusion MRI study. Brain.

[12] Radbruch A, Weberling LD, Kieslich PJ (2015). Gadolinium retention in the dentate nucleus and globus pallidus is dependent on the class of contrast agent. Radiology.

[13] McDonald RJ, McDonald JS, Kallmes DF (2015). Intracranial Gadolinium Deposition after Contrast-enhanced MR Imaging. Radiology.

[14] Kanda T, Fukusato T, Matsuda M (2015). Gadolinium-based Contrast Agent Accumulates in the Brain Even in Subjects without Severe Renal Dysfunction: Evaluation of Autopsy Brain Specimens with Inductively Coupled Plasma Mass Spectroscopy. Radiology.

[15] Kanda T, Ishii K, Kawaguchi H, Kitajima K, Takenaka D (2014). High signal intensity in the dentate nucleus and globus pallidus on unenhanced T1-weighted MR images: relationship with increasing cumulative dose of a gadolinium-based contrast material. Radiology.

[16] Debernard L, Melzer TR, Van Stockum S (2014). Reduced grey matter perfusion without volume loss in early relapsing-remitting multiple sclerosis. J Neurol Neurosurg Psychiatry.

[17] Gardener AG, Gowland PA, Francis ST (2009). Implementation of quantitative perfusion imaging using pulsed arterial spin labeling at ultra-high field. Magn Reson Med.

[18] Teeuwisse WM, Webb AG, van Osch MJ (2010). Arterial Spin Labeling at Ultra-High Field: All That Glitters is Not Gold. Int J Imaging Syst Technol..

[19] Hall EL, Wesolowski R, Gowland PA, Francis ST (2010) Optimising image readout for perfusion imaging at 7T. Proceedings of the 18th Annual Scientific Meeting and Exhibition of the International Society for Magnetic Resonance in Medicine, Stockholm, 1–7 May 2010

[20] Samaraweera AP, Clarke MA, Whitehead A (2017). The Central Vein Sign in Multiple Sclerosis Lesions Is Present Irrespective of the T2* Sequence at 3 T. J Neuroimaging.

[21] Hurley AC, Al-Radaideh A, Bai L (2010). Tailored RF pulse for magnetization inversion at ultrahigh field. Magn Reson Med.

[22] Jenkinson M, Bannister P, Brady M, Smith S (2002). Improved Optimisation for the Robust and Accurate Linear Registration and Motion Correction of Brain Images. Neuroimage.

[23] Smith SM (2002). Fast robust automated brain extraction. Hum Brain Mapp.

[24] Maumet C, Maurel P, Ferré JC, Barillot C (2014). Robust estimation of the cerebral blood flow in arterial spin labelling. Magn Reson Imaging.

[25] Blockley NP, Jiang L, Gardener AG, Ludman CN, Francis ST, Gowland PA (2008). Field strength dependence of R1 and R2* relaxivities of human whole blood to ProHance, Vasovist, and deoxyhemoglobin. Magn Reson Med.

[26] Francis ST, Bowtell R, Gowland PA (2008). Modeling and optimization of Look-Locker spin labeling for measuring perfusion and transit time changes in activation studies taking into account arterial blood volume. Magn Reson Med.

[27] Dury RJ, Falah Y, Gowland PA, Evangelou N, Francis ST, Bright MG (2018) Reproducibility and quality assessment of a 3D-EPI pulsed arterial spin labelling scheme at 7 T in a clinical cohort, (abstract) Proc Intl Soc Magn Reson Med Ann Mtg Paris, France

[28] Gardener AG, Jezzard P (2015). Investigating white matter perfusion using optimal sampling strategy arterial spin labeling at 7 Tesla. Magn Reson Med.

[29] D'Ortenzio RM, Hojjat SP, Vitorino R (2016). Comparison of Quantitative Cerebral Blood Flow Measurements Performed by Bookend Dynamic Susceptibility Contrast and Arterial Spin-Labeling MRI in Relapsing-Remitting Multiple Sclerosis. AJNR Am J Neuroradiol.

